# Single Cell Profiling Reveals *PTEN* Overexpression in Influenza-Specific B cells in Aging HIV-infected individuals on Anti-retroviral Therapy

**DOI:** 10.1038/s41598-019-38906-y

**Published:** 2019-02-21

**Authors:** Lesley R. de Armas, Suresh Pallikkuth, Li Pan, Stefano Rinaldi, Nicola Cotugno, Sarah Andrews, Rajendra Pahwa, Adrian B. McDermott, Paolo Palma, Savita Pahwa

**Affiliations:** 1 University of Miami, Miller School of Medicine, Department of Microbiology and Immunology, Miami, Florida USA; 2 Academic Department of Pediatrics (DPUO), Research Unit in Perinatal Infections, Children’s Hospital Bambino Gesù, Rome, Italy; 3 Department of Public Health, University of Rome Tor Vergata, Rome, Italy; 4 Vaccine Research Center, NIAID-NIH, Bethesda, MD USA

## Abstract

Memory B cells (MBC) respond to secondary antigen challenge to protect against infection and to boost immunity following vaccinations. Despite effective treatment, chronic HIV infection disturbs MBCs by reducing numbers and altering functionality due to hyper-activation and increased apoptosis leading to suboptimal antibody responses against common infectious agents. We used single cell gene expression analysis to evaluate antigen-specific memory B cells in peripheral blood of virally-suppressed HIV-infected individuals and healthy controls stratified by serum H1N1 antibody response 3 weeks post-administration of the seasonal trivalent inactivated influenza vaccine. We used a fluorescent probe to isolate influenza H1N1-specific B cells and a multiplexed and targeted RT-PCR approach to measure expression levels of 96 genes involved in B cell activation and function. Gene profiling revealed a 4-gene predictive signature containing the phosphoinositide-3 kinase (PI3K) inhibitor, *PTEN*, for identifying antigen-specific MBC from HIV-infected individuals compared to healthy controls. Gene co-expression analysis showed that in addition to overexpression of *PTEN*, there was increased co-expression of type I interferon-associated genes with *PTEN* on single cell level in HIV compared to controls. This study highlights the persistent defects in MBC from HIV-infected individuals and points to the PI3K signaling pathway as a target for potential immune intervention.

## Introduction

Memory B cells (MBC) are an important component of the immune system which are maintained for long periods following induction by vaccination or infection. Classically defined MBCs express class-switched, somatically hyper-mutated (SHM) B cell receptors (BCR) following a germinal center (GC) reaction. MBC make up approximately 40% of all B cells in human adults and are a highly diverse population including IgG+, IgA+, and IgM + isotype populations^1^. Single MBC clones derived from a GC reaction can include more than one isotypic subset, demonstrating the functionally heterogeneous nature of these cells. Further, circulating MBC can be delineated phenotypically by varying expression of the surface markers CD27 and CD21 whereby the majority of MBC are identified as resting memory (RM, CD27+ CD21+) followed by activated memory (AM, CD27 + CD21 low/neg) and “tissue-like” memory (TLM, CD27 low/neg CD21 low/neg)^2^. The MBC compartment is critical for response to infection and is therefore a target for vaccine development against pathogens, including human immunodeficiency virus (HIV). Broadly neutralizing anti-HIV antibodies (bNabs) have been isolated from HIV patients, following years of antigen exposure and many rounds of affinity maturation and SHM. These isolated bNabs are under investigation for passive immune prophylaxis and therapeutic intervention^3^. During uncontrolled viremia, B cells producing anti-HIV antibodies have an altered phenotype compared to anti-influenza antibody producing B cells within individual patients^4,5^. Although B cell defects, including cell turnover, hyper-activation and increased apoptosis are reverted with ART initiation, MBC impairment remains^6^ due to chronic immune activation attributed to persistence of HIV antigen in lymph nodes and other sanctuary sites^7–10^.

Seasonal influenza vaccination is a useful modality for investigating immune response^11,12^. Following vaccination, influenza-specific B cells expand, peaking around 7 days post-vaccination, and remain elevated up to one month post-vaccination^13^. Increase in serum titers of anti-influenza antibodies is a measure of immune response to the vaccination. We have previously shown that influenza-specific responses in B cells^14,15^, T cells^16–18^, and the innate immune system^19^ are impaired in HIV-infected individuals in the context of viral suppression by ART in both young and old (>60 years) individuals. However, these studies have largely been performed using bulk cell analysis from *in vitro* antigen-stimulated culture experiments. Technological advances in single cell analysis allow for deeper interrogation of cellular states in cell populations with diverse functions, such as MBC. Here, we used a single cell, targeted multiplex gene expression platform and predictive modeling to show that following *in vivo* stimulation with the seasonal flu vaccine, influenza-specific MBC exhibit divergent gene signatures in HIV-infected, ART-suppressed individuals compared to age-matched healthy controls (HC). The resulting gene signature implicates PTEN-mediated inhibition of PI3K signaling pathway as a key player in persistent B cell dysfunction during HIV infection thereby providing a potential target for intervention in improving vaccine-induced antibody responses.

## Results

### Reduced memory B cell responses to influenza vaccination in HIV-infected individuals

**Figure 1:**
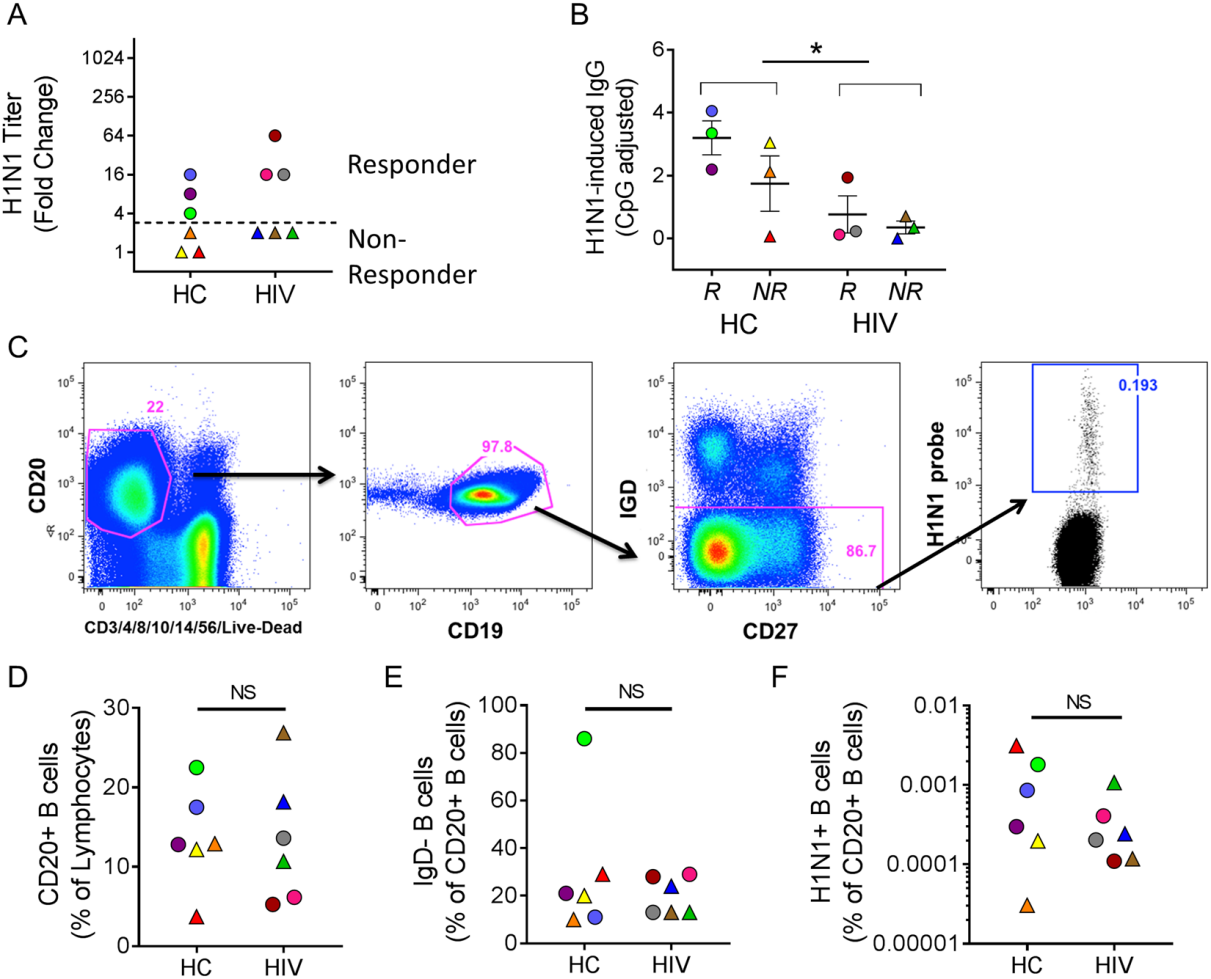
Serologic and phenotypic characterization of *ex vivo* B cells after influenza vaccination. (**A**) Fold change (3 wks post-vaccination/baseline) H1N1 HAI titer for each individual. The dotted line shows the threshold for discriminating between responders and non-responders. (**B**) B cell Elispot data from 3 wks post-vaccination showing IgG production in response to H1N1 stimulation for 5 days. *denotes p < 0.05 after t test comparing HC and HIV groups. (**C**) Gating scheme for isolating H1N1-specific B cells. Frequency of (**D**) CD20+ B cells, (**E**) IgD- B cells, and (**F**) H1N1-specific B cells in study participants.

12 individuals were selected from a cohort of HIV-infected and healthy control adult volunteers (age range 60–76 yrs.) participating in an influenza vaccination study (FLORAH cohort)^15^ to evaluate gene profiles of *ex vivo* H1N1-specific B cells (Table 1). All HIV-infected participants were virologically suppressed on ART. The H1N1 serum titers in this cohort are shown in Supplemental Fig. 1. Vaccine responders were defined as individuals that showed at least 4-fold increases in H1N1 antibody titers 3 weeks post-vaccination. In the HC group 23/51 (45%) were classified as responders while in HIV group only 16/50 (32%) were H1N1 responders. This distribution of responders (R) and non-responders (NR) is similar to other influenza vaccination studies^18,20^. Participants were excluded in this selection if they had high baseline titers against H1N1 (>1:80) and we selected an equal number of responders and non-responders to allow for comparison by serological response to vaccination. The fold increases in serum titer to H1N1 are shown (Fig. 1A). HIV R exhibited a trend of higher fold increase compared to HC R although they exhibited an overall lower number of H1N1 specific memory B cells as determined by H1N1 IgG ELISpot (Fig. 1B). H1N1-specific B cells were identified using a panel of monoclonal antibodies and a fluorescently-tagged H1N1 probe^21^ (Fig. 1C). HIV status did not affect the frequencies of CD20+ cells, IgD negative cells, or H1N1-specific B cells (Fig. 1D–F). Vaccine response status also showed no relationships with B cell subset frequency measurements in the participants.

**Table 1:** Study Participants.

Gender	HIV status	Age (yrs.)	H1N1 Serum Titer			Response Status
			T0	T2	T2/T0	
Male	Negative	62	10	10	1	Non-Responder
Male	Negative	66	20	40	2	Non-Responder
Female	Negative	67	80	80	1	Non-Responder
Male	Negative	60	40	160	4	Responder
Female	Negative	61	40	640	16	Responder
Female	Negative	66	40	320	8	Responder
Male	Positive	67	20	40	2	Non-Responder
Male	Positive	66	40	80	2	Non-Responder
Male	Positive	62	40	80	2	Non-Responder
Female	Positive	62	10	160	16	Responder
Male	Positive	60	10	160	16	Responder
Male	Positive	76	20	1280	64	Responder

### PI3K-inhibitor *PTEN* is overexpressed in H1N1-specific memory B cells in HIV

**Figure 2:**
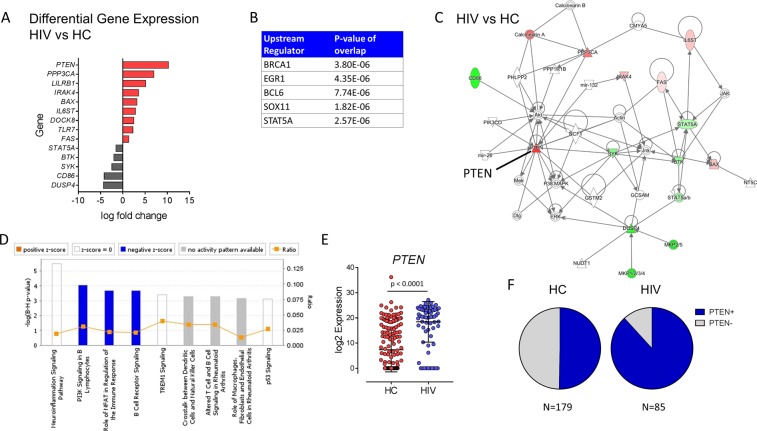
Transcriptional signatures of H1N1-specific B cells in HIV-infected individuals compared to Healthy controls. (**A**) Log fold change expression of differentially expressed genes in single cells from HIV and healthy controls (HC) with FDR-adjusted p value < 0.05 using MAST analysis. Red bars indicate higher expression in HIV and gray bars indicate higher expression in HC. (**B**) Upstream regulator analysis (**C**) Network analysis and (**D**) Pathway analysis using Ingenuity Pathway Analysis software shows top results using the 14 differentially expressed genes between HIV and HC identified in A. In C, Red color indicates higher expression in HIV-infected and green color indicates lower expression in HIV. (**E**) Scatter plot showing log 2 expression levels of PTEN gene expression for each single cell from HC (Red) and HIV (blue). (**F**) Pie charts showing proportion of cells with detectable *PTEN* expression in HIV and HC.

Individual IgD-negative H1N1-specific B cells were sort-purified into 96 well plates and evaluated for transcript expression of a previously validated gene panel designed to investigate the activation status and functionality of B cells in response to vaccination using the Fluidigm BioMark platform^22^. The panel included genes related to B cell receptor signaling, antibody production, cytokine signaling, immune regulation, cell activation and proliferation, and interferon responses (Supplementary Table S1). MAST package^23^ was used to analyze RT-PCR data and identified 14 differentially expressed genes (DEG) between H1N1-specific B cells from HC and HIV-infected participants. 9 out of 14 genes showed greater expression in single cells from HIV compared to HC including *PTEN*, *PPP3CA*, *LILRB1*, *IRAK4*, *BAX*, *IL6ST*, *DOCK8*, *TLR7*, and *FAS* while 5 genes were downregulated including *STAT5A*, *BTK*, *SYK*, *CD86*, and *DUSP4* (Fig. 2A). Ingenuity Pathway analysis (IPA, Qiagen Bioinformatics) standard workflow was applied to evaluate direct relationships to further evaluate these 14 DEGs in relation to predicted a) upstream transcriptional regulators, b) interaction networks, and c) altered signaling pathways that may be involved. The upstream regulators implicated in control of the observed differential gene expression included BRCA1, EGR1, BCL6, STAT5A, and SOX11 though the direction of the activation was not able to be determined (Fig. 2B). The top interaction network generated in analysis of the 14 DEGs points to an activated profile in HIV compared to HC with *PTEN* overexpression and *SYK* downregulation playing a central role due to their positions in the network (Fig. 2C). *PTEN* is an inhibitor of PI3K, a member of the PI3K/AKT/mTOR signaling pathway which plays major roles in cellular metabolism and regulating immune functions; therefore it was not surprising to find significant alterations in the following signaling pathways ‘PI3K signaling in B lymphocytes’, ‘Role of NFAT in Regulation of the Immune Response’, and ‘B Cell Receptor Signaling’ which were all decreased in cells from HIV-infected individuals (Fig. 2D). High *PTEN* expression in H1N1-specific B cells from HIV was due to increases in continuous (Fig. 2E) and discrete expression with 88% of cells having detectable *PTEN*, meanwhile in HC, *PTEN* was detectable in 50% of B cells (Fig. 2F).

### Distribution and gene expression are altered in memory B cell subsets in HIV

**Figure 3:**
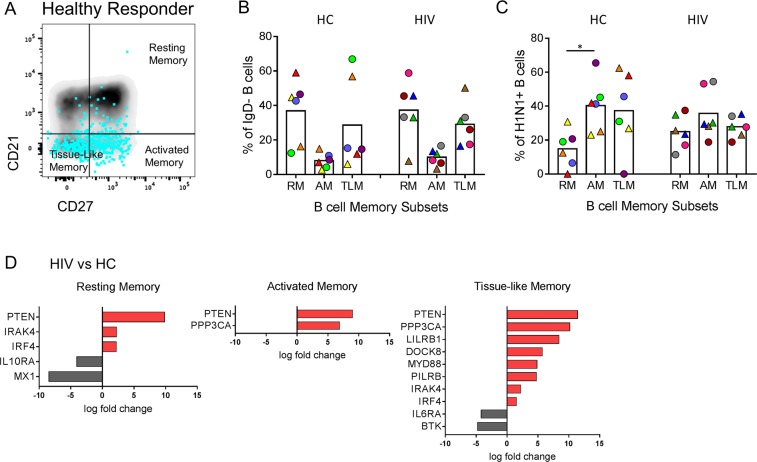
Memory B cell subset distribution and gene expression for H1N1-specific B cells. (**A**) The distribution of CD21 and CD27 expression on the surface of sorted H1N1-specific B cells (blue dots foreground) relative to total B cells (density plot background) is shown from a representative Healthy Control (HC). (**B**) Memory cell distribution as a frequency of IgD- B cells is shown for HC and HIV participants. (**C**) Memory cell distribution as a frequency of H1N1-specific B cells is shown for HC and HIV participants. (**D**) Log fold change expression of differentially expressed genes in single cells from HIV relative to HC in each memory B cell subset using MAST analysis (FDR-adjusted p value < 0.05).

To determine whether the observed gene profiles were confined to a specific maturation phenotype within the sorted H1N1-specific B cells from HC and HIV, we evaluated indexed flow cytometry data collected at the time of sorting for CD27 and CD21 expression on single cells. Using these 2 surface markers we were able to distinguish 3 memory phenotypes: resting memory (RM, CD27+ CD21+), activated memory (AM, CD27+ CD21−), and tissue-like memory (TLM, CD27− CD21−) for each group (Fig. 3A). Subset distributions within the total B cell memory pool were determined for each participant and the majority of cells were found between the RM and TLM subsets (Fig. 3B). H1N1-specific B cells on the other hand, had low frequencies of RM and were enriched in AM in HC. In HIV, H1N1-specific B cells were equally distributed across all subsets (Fig. 3C). Direct comparison of subset frequencies between HC and HIV did not yield significant differences. Next, we evaluated gene expression between HIV and HC in each of the memory subsets to determine contributions to the gene signature from each subset. *PTEN* expression was not confined to a particular subset in H1N1-specific B cells from HIV, as it displayed the highest fold change increase compared to HC B cells in all memory subsets (Fig. 3D). TLM cells exhibited the greatest contrast between HIV and HC with 10 DEGs including *PTEN* and other shared genes with RM (*IRAK4* and *IRF4)* and AM phenotypes (*PPP3CA)*. TLM from HIV also showed higher *LILRB1*, *MYD88*, *DOCK8*, *PILRB* and lower *IL6RA* and *BTK* compared to HC. RM from HC had higher expression of *IL10RA* and *MX1* compared to HIV. Overall these data demonstrate that B cells specific to a non-HIV antigen show alterations at the gene transcriptional level that are not discernible at the level of surface marker expression and that transcriptional alterations affect all memory subsets.

### *PTEN* is co-expressed with IFN-inducible genes in H1N1-specific cells from HIV

An advantage of single cell transcript analysis is the possibility to evaluate co-expression of multiple genes at a single cell level similar to flow cytometry analysis rather than averaging data from bulk cell preparations. Given the strong overexpression of *PTEN* in H1N1-specific cells from HIV, we analyzed co-expression of *PTEN* with all other genes in the panel. This analysis revealed *BTK*, *PPP3CA*, and *TLR7* with positive correlations in HC participants only. *PTEN* correlated positively with 4 additional genes regardless of HIV status including *APOBEC3G*, *IGM*, *IRAK4*, and *STAT5A* suggesting that the latter genes are tightly co-regulated with *PTEN* expression (Table 2). Additional genes that were co-expressed with *PTEN* only in HIV included *BST2*, *DOCK8*, *IFNAR2*, *IL10RA*, and *NFKB1*. These results indicate that *PTEN* associated signaling pathways are perturbed in B cells from HIV-infected individuals despite viral suppression with cART.

**Table 2:** Correlation of gene expression with *PTEN* in H1N1-specific B cells.

Target Gene	Query Gene	Healthy Control		HIV	
		Pearson Coefficient	P value	Pearson Coefficient	P value
*PTEN*	*APOBEC3G*	0.18	**0**.**02**	0.37	**0**.**001**
*PTEN*	*IGM*	0.45	<**0**.**0001**	0.32	**0**.**004**
*PTEN*	*IRAK4*	0.52	<**0**.**0001**	0.34	**0**.**002**
*PTEN*	*STAT5A*	0.34	<**0**.**0001**	0.27	**0**.**02**
*PTEN*	*BTK*	0.21	**0**.**009**	0.13	0.26
*PTEN*	*PPP3CA*	0.21	**0**.**01**	0.01	0.92
*PTEN*	*TLR7*	0.27	**0**.**001**	0.08	0.48
*PTEN*	*BST2*	0.03	0.67	0.26	**0**.**02**
*PTEN*	*DOCK8*	−0.04	0.65	0.34	**0**.**003**
*PTEN*	*IFNAR2*	0.13	0.12	0.25	**0**.**03**
*PTEN*	*IL10RA*	0.07	0.41	0.42	**0**.**0001**
*PTEN*	*NFKB1*	0.05	0.56	0.24	**0**.**04**

### Ig Isotype expression in H1N1 specific B cells

**Figure 4:**
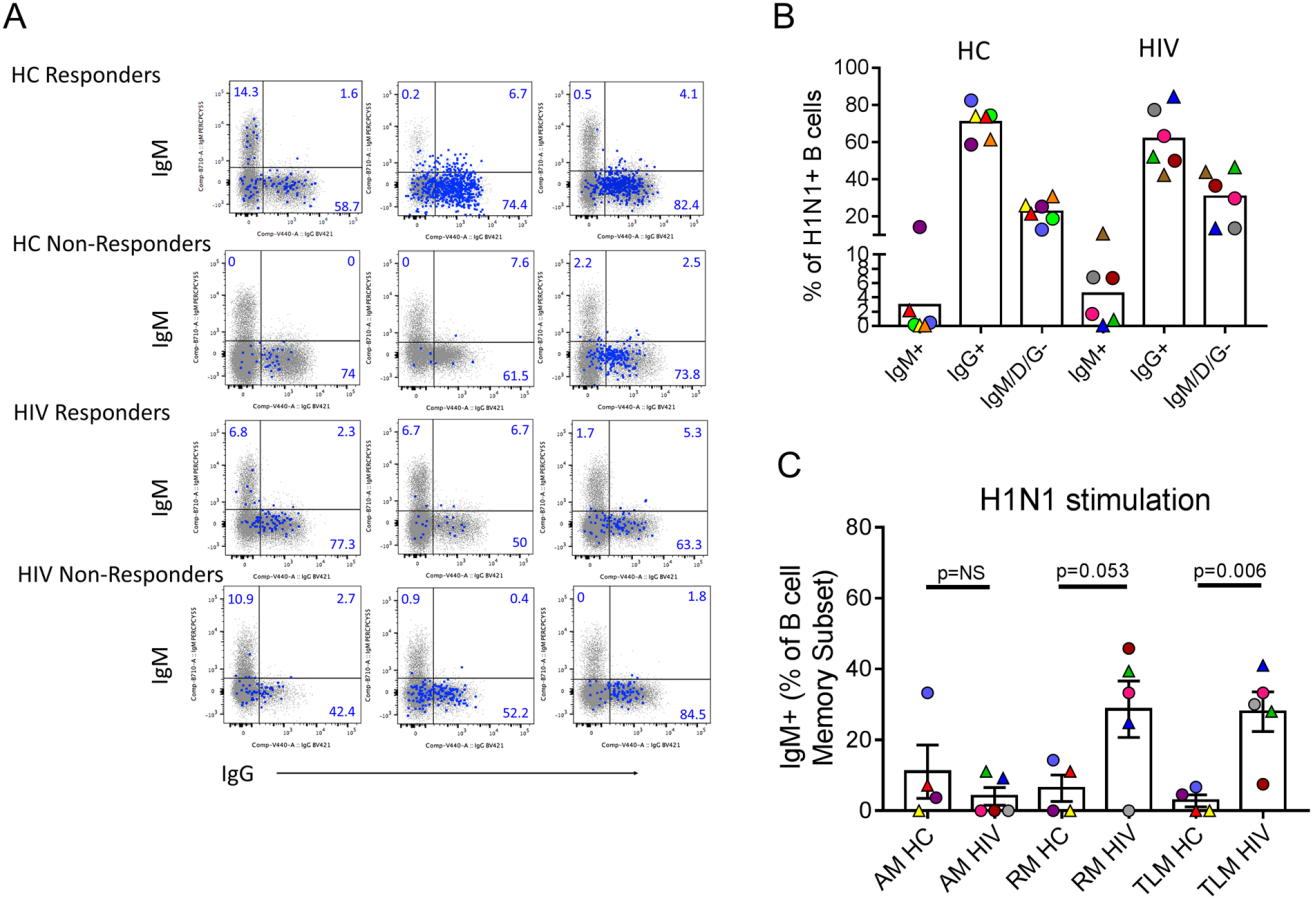
Memory B cell Ig Isotype expression of H1N1-specific B cells. (**A**) Overlay dot plots showing Ig isotype distribution of IgM (y-axis) and IgG (x-axis) for all study participants in total IgD- B cells (small gray dots, underlay) and H1N1-specific IgD- B cells (large blue dots, overlay). Numbers in quadrants refer to frequency of cells in H1N1-specific population. (**B**) Summary of data for H1N1-specific B cells from (**A**) is shown for HC and HIV participants. (**C**) Frequencies of IgM memory B cells (IgD− fraction) within each subset are shown for HC and HIV participants following 5 day *in vitro* culture of PBMC with H1N1 antigen. Student’s t test was performed between groups indicated, **denotes.

To follow up on the *PTEN: IgM* co-regulation, we analyzed Ig isotype expression measured at the time of single cell sorting from total memory and H1N1-specific cells by flow cytometry (Fig. 4A). H1N1-specific cells from both participant groups were dominated by IgG expression, though in HIV 3/6 participants had a detectable IgM + population whereas in HC only 1/6 had detectable IgM memory cells (Fig. 4B). In a separate experiment, PBMC from study participants were stimulated *in vitro* with H1N1 antigen for 5 days and surface expressed IgM and IgG were measured by flow cytometry. RM and TLM B cell subsets in HIV showed higher expression of IgM-expressing cells compared to HC (Fig. 4C). No significant difference in frequency of IgG producing cells after antigen stimulation were observed (not shown).

### *PTEN* drives a predictive signature able to discriminate between H1N1-specific Memory B cells from HIV and HC

**Figure 5:**
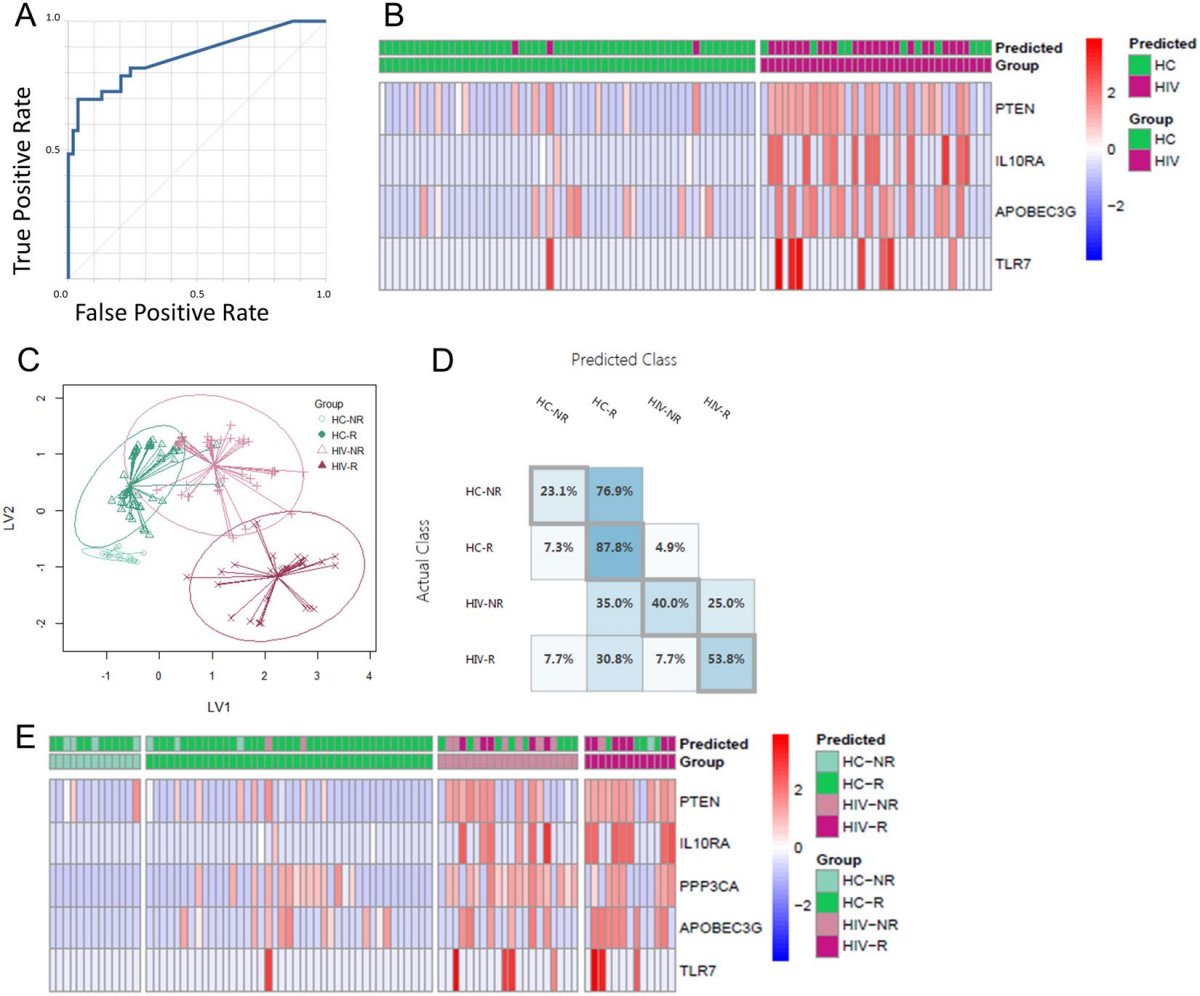
Predictive modeling of H1N1-specific B cells. For classification of HIV vs HC, (**A**) Receiver operating characteristic (ROC) curve for two-class neural network showing prediction accuracy of 0.864. Single cell gene expression data for 33 genes was split 2:1 for train: test. (**B**) Z score heat map (scaled by gene) showing ‘test’ set of single cells with prediction and actual grouping based on 4-gene signature. For classification of Vaccine Responder and Non-Responder for HIV and HC: (**C)** Graphical representation of LASSO-PLSDA model using 5-gene signature to segregate 4 groups: HC R, HC NR, HIV R, HIV NR. (**D)** Classification matrix for the 4 groups showing performance of the model by prediction accuracy for each group. (**E)** Z score heat map (scaled by gene) showing ‘test’ set of single cells with prediction and actual grouping based on 4-gene signature.

To explore the strength of the HIV-specific gene signature observed in H1N1-specific B cells we applied machine-learning analysis methods. We used 33 differentially expressed genes identified between subpopulations (Figs 2A, 3D and S2) to build a machine-learning predictor to identify B cells from HIV infected individuals. First, LASSO^16,24^, a variable selection regression model, was employed to avoid over-fitting the model due to co-expression of genes. We used four machine learning algorithms and cross-validation by 3-, 5-, and 10-fold to estimate the predictive accuracy for identifying a cell from either group. Each algorithm resulted in AUC values greater than 0.83 and the highest was 0.89 (Fig. 5A and Supplemental Table S2). This analysis resulted in a 4-gene signature with high predictive accuracy (0.851) and precision (0.885) that included *PTEN*, *IL10RA*, *APOBEC3G*, and *TLR7* (Fig. 5B).

To determine the effects of altered gene profiles in B cells from HIV-infected, cART-suppressed participants on their response to vaccination, we used machine learning to classify single cells into 4 groups by HIV status and serological response to the seasonal flu vaccine (HC R, HC NR, HIV R, and HIV NR). We used the same 33 differentially expressed genes identified between the subpopulations, including those identified from comparison between R and NR in each group separately (Supplemental Fig. S3). The test group was plotted using PLS-DA and showed distinct clustering of the 4 groups (Fig. 5C). However, the predictive accuracy was reduced for the 4 group classification compared to the 2 group with an average of 0.62 for the AUC. HC R showed the highest accuracy out of all 4 groups while HC NR showed the lowest (Fig. 5D). The resulting signature included the same 4 genes from the HIV/HC classification model but included *PPP3CA* as an additional gene, thus clearly being dominated by the gene differences between HIV and HC rather than between R and NR (Fig. 5E).

## Discussion

In this study we used single cell gene expression analysis to evaluate transcriptional variation that can influence antigen-specific memory B cell functionality in the context of vaccine-induced antibody responses and chronic HIV infection. The driving question was derived from the observation that current biomarkers of influenza vaccine response (i.e. serum titers) are not fully informative especially in populations with acquired immune deficiencies^25^. Hence, novel correlates of functional memory B cell responses are needed. Single cell analysis allowed us to obtain robust and significant data from a small number of individuals and cells which were otherwise phenotypically indistinguishable by flow cytometry-based cell surface protein measurements. Although single cell RNA-Seq technology is becoming widely-accessible and more cost effective using a variety of instruments and commercially available reagents for library preparation, the multiplex targeted gene expression allows one to interrogate specific genes of interest. A limitation of whole transcriptome (i.e. RNA-Seq) applications with resting lymphocytes is the efficiency for detection of the relatively small amount of RNA present in the cell (compared to cell lines for example) can be at or near the lower limit of detection for low-depth sequencing techniques. Thus, the targeted approach allows for assessment of select gene expression without the cost of ultra-deep sequencing per cell.

Single cell gene expression data revealed overexpression of *PTEN* in H1N1-specific memory B cells from HIV-infected individuals. This is the first time *PTEN* has been directly connected with human B cell dysfunction outside of the cancer field where *PTEN* is a well-described tumor suppressor gene whose mutation or deletion plays a role in a variety of sporadic human cancers^26^. *PTEN* has been shown to dampen BCR responses through direct inhibition of PI3K in situations of self-reactivity^27^ and virus infection using murine models^28^. PI3K signaling along with BCR engagement is required to induce the activation of B cells and their maturation toward antibody secreting cells^29^. Genetic mutations in PI3K can lead to primary immune deficiency such as the recently described activated PI3 kinase delta syndrome, which leaves patients susceptible to recurrent respiratory tract infections^30^. Our results suggest that *PTEN* over-expression is experienced globally by antigen-specific memory B cells during HIV infection rather than an isolated effect on HIV-specific immunity due to chronic antigenic stimulus as would have been predicted by previous studies in mice^28^. Acute viral infection often leads to polyclonal B cell activation, therefore suppression of the humoral immune system via PI3K inhibition during this period could be a host mechanism to prevent bystander activation of self-reactive B cells. The molecular mechanisms governing *PTEN* overexpression in H1N1-specific B cells from this study are unknown, however positive regulators of *PTEN* gene expression include early growth response protein-1 (EGR-1), peroxisome proliferator-activated receptor-γ (PPAR-γ), tumor protein 53 (p53), and human sprout homolog 2 (SPRY2)^31^. Interestingly, EGR-1 is a transcription factor broadly involved in cell survival and was identified as an upstream regulator in pathway analysis of DEGs between HIV and HC (Fig. 2C), and thus may be responsible for *PTEN* expression observed. BCL6 was also identified as a likely regulator of the transcriptional differences between HIV and HC H1N1-specific single cells. BCL6 is expressed at high levels in germinal center B and T follicular helper cells^32^. In B cells, cell migration and differentiation from mature B cells into plasma cells is controlled by the switch from BCL6 driven transcriptional program to the antagonistic BLIMP1 program^33^.

Single cell gene co-expression analysis revealed that *PTEN* expression in H1N1-specific B cells positively correlated with *IGM* gene expression (Table 2). We also noted more participants with detectable IgM-expressing H1N1-specific B cells in HIV infected individuals. The presence of IgM memory in response to influenza vaccination in HIV-infected individuals might explain the discrepancy between high serum titers against H1N1 but reduced IgG Elispot results observed in the HIV-infected vaccine responders since the HAI reaction used to quantify serum titers cannot distinguish between Ig isotypes. IgM-secreting MBC share many similarities with IgG-secreting MBC including accumulation with age and the presence of *BCL6* mutations due to co-expression with activation-induced cytidine deaminase (AID) during a GC reaction^34^. However, the function of IgM  memory remains controversial; some argue that IgM MBC re-enter GC upon secondary re-challenge and IgG MBC preferentially differentiate into antibody secreting plasma cells^35^ while other studies have shown that IgM MBC are early and potent responders to antigen re-challenge resulting in robust antibody secretion in response to malaria^36^. The impact of influenza-specific IgM memory cells on overall immunity against influenza infection is not yet understood though the observed increase in this population in HIV participants in this study suggests they may play a negative or compensatory role in HIV-infected individuals. Interestingly, co-expression analysis in this study showed associations between the expression of *PTEN* in H1N1-specific Memory B cells in HIV with type I Interferon-associated genes such as *BST2* (tetherin) and *IFNAR2* indicating the possibility that *PTEN* over-expression could be linked to reduced functional antibody responses in these cells via the type I interferon response pathway.

Modeling the four participant groups using single cell gene expression data allowed us to appreciate the functional gap between HC responders and HIV responders, and gene profiles associated with non-responders were equally divergent between HC and HIV. These data indicate that despite the presence of ART, the alterations induced by HIV chronic infection on B cell antigen-specific responses persist and are of sufficient magnitude that necessitates separate analysis of HIV individuals from their healthy peers. Other studies addressing the effects of HIV on B cell function during ART have shown persistent defects in the form of reduced numbers of MBC and altered expression of cell surface markers related to activation and apoptosis^37,38^. In this study when HIV infected individuals were analyzed separately to compare R and NR, B and T Lymphocyte Associated (*BTLA)* expression was found to be higher in HIV NR compared to R, whereas all other DEGs showed higher expression in R (Fig. S4). Elevated *BTLA* suggests a role for immune regulatory mechanisms in inhibiting vaccine-induced antibody responses, similar to what has been observed in T cells with PD-1 and other immune checkpoint molecules during immune exhaustion^39^.

The predictive signature for identifying H1N1-specific B cells from HIV-infected individuals was small containing only 4 genes, including *PTEN*, *IL10RA*, *APOBEC3G*, and *TLR7*. The role of IL10 in B cells is thought to be regulatory, as it is for T cells and other components of the immune system, and it has also been shown to promote IgM production and suppress apoptosis^40^. *APOBEC3G* is a host restriction factor that plays an important role by interfering with reverse transcription during HIV replication in CD4 T cells during HIV replication. It is expressed in other cell types including B cells where co-expression with AID can affect somatic hypermutation and antibody maturation^41,42^. The final gene in the predictive signature, *TLR7* is an endosomal toll- like receptor recognizing ssRNA, and in naïve B cells provides a third signal for activation. TLR7 ligation in B cells promotes class-switching to IgG^43^. Both *APOBEC3G* and *TLR7* are induced by type I interferon, suggesting that the presence of an ongoing anti-viral response in the HIV-infected subjects is shaping the type of antibodies produced in response to vaccination. Overall, this signature reinforces the concept that enhanced type I interferon signaling can lead to impairment in B cell maturation where PTEN seems to play a central role. Further, this study provides a framework for analysis of antigen-specific cells using single cell gene expression analysis and provides insight into persistent defects in B cell-mediated immunity in the context of treated HIV infection and introduce potential targets of intervention to improve vaccine responses.

## Methods

### Human PBMC Samples

Study participants were enrolled in a prospective, open-label Influenza Vaccine study and were recruited from University of Miami, Jackson Memorial, and VA Hospitals in Miami, FL. Participants were recruited during the 2014–15 Flu season from two populations: (1) HIV-infected, ART-treated individuals who demonstrated virus suppression (HIV RNA <40 copies/ml) for at least 1 year prior to enrollment, and (2) HIV-uninfected healthy controls. Peripheral blood was collected from participants at pre-vaccination (T0) and day 21 post-vaccination (T2) with influenza virus vaccine, trivalent, types A and B (TIV). Peripheral blood mononuclear cells (PBMC) and plasma were stored in liquid nitrogen and −80C freezers, respectively, until further experiments were performed.

This study was approved by the Institutional Review Boards of University of Miami and Miami Veterans Affairs Medical Center and was carried out in accordance with approved guidelines. Voluntary signed informed consent was obtained from every participant prior to participating in the study.

### Determination of B cell response to H1N1 vaccination

#### Hemagluttination Inhibition (HAI) Assay

Serum Ab titers to H1N1 influenza strains were evaluated separately using an HAI assay, which was performed as previously described (21). HAI titers are expressed as the reciprocal of the highest serum dilution at which hemagglutination was prevented.

#### ELISPOT Assay

H1N1-specific memory B cells (MBC) were measured from T2 by ELISPOT assay as described^14^. For MBC enumeration, previously cryopreserved PBMC were thawed, rested, and stimulated with 5 µg/mL H1N1/09 vaccine antigen plus anti-CD28 mAb (1 µg/mL), CpG (as a positive control) or unstimulated (as negative control) for 5 days at 37 °C. On day 5, cells were plated in wells coated with goat anti-human IgG (2 µg/mL, Jackson Immunoresearch) at 100,000 cells/well for 4 hours at 37 °C and assayed for H1N1-specific IgG. Spots were read using Immunospot software and plate reader (Cellular Technologies LTD). H1N1-specific IgG results were normalized and graphed as proportion of the positive control.

### *In vitro* PBMC Stimulation

Cryopreserved PBMC from T2 were thawed and rested overnight before culturing for 5 days with 5 µg/mL H1N1/09 vaccine antigen plus anti-CD28 mAb (1 µg/mL), CpG (as a positive control) or unstimulated (as negative control) at 37 °C. Cells were labeled with fluorescently labeled monoclonal antibodies against surface molecules CD20, CD19, CD27, CD21, CD38, CD10, IgD, IgM, and IgG. Dead cells were excluded from analysis using Live/Dead Fixable Aqua (Thermofisher). Cells were acquired on and LSRII instrument (BD Immunocytometry systems) and analyzed by FlowJo Software (TreeStar).

### FACS Sorting

Cryopreserved PBMC samples were thawed and rested before staining with monoclonal antibodies against CD20, CD19, CD27, CD21, CD38, IgD, IgG, and IgM. Dead and non-B cells were excluded from analysis using a cocktail of antibodies against CD3, CD4, CD8, CD10, CD14, CD56, and Live-Dead stain in the same fluorescent channel (Dump channel). The HA probe specific for H1N1 was generated and conjugated for flow cytometry as previously described^21^ and was included in the antibody staining master mix at a pre-titrated concentration. The samples were acquired on the same day using an LSRII instrument (BD Immunocytometry Systems). One to two million events were collected per sample and analyzed using FlowJo software (TreeStar). For cell sorting, 96 live Dump− CD20 + CD19 + IgD− H1 + cells were sorted into a 96-well plate containing 9ul CellsDirect (Thermofisher) RT-PCR amplification buffer and reagents as previously described^22^.

### Single cell RT-PCR

After sorting cells were immediately spun down and kept on ice briefly prior to performing one-step reverse transcription and cDNA amplification of 96 specific targets (Supplemental Table S1) using a pool of Taqman^TM^ gene expression assays (Thermofisher) using the following protocol: 50 °C for 20 min, 95 °C for 2 min, 95 °C for 15 s, and 60 °C for 4 min; the last two steps were repeated for 21 cycles. Resulting cDNA was loaded onto BioMark IFC 96 × 96 chip (Fluidigm) according to the manufacturer’s protocol. A total of 360 H1N1-specific B cells were sorted from 12 participants (range from a single individual 4–59) (Supplemental Fig. S2). We utilized the MAST package, a statistical framework using the hurdle model, which accounts for bimodality of gene expression by simultaneously accounting for expression rate (discrete) and average expression (continuous) values^23^. Initial analysis showed a bimodal distribution of expression for most genes (Supplemental Fig. S2). Raw data underwent ‘cellular detection rate’ (CDR) filtering to remove outlier samples and genes based on dataset distribution^23,44^. *CD74* (also known as *HLADG*) was used as a surrogate for the presence of a cell (i.e. loading control) due to its stable expression in lymphocytes. Cells that had low or absent *CD74* expression exhibited reduced gene expression globally and were removed from analysis. (Supplemental Fig. S2). Overall 96 cells and 36 genes were filtered out following quality control sanitation steps and differential gene expression analysis was subsequently performed to contrast transcriptional profiles of H1N1-specific B cells between HC and HIV-infected participants.

### Pathway Analysis

Differential gene expression data was loaded along with fold change and p-values for comparison between HIV and HC into Ingenuity Pathway Analysis software. Only direct relationships were included in the analysis using the Ingenuity Knowledge base (genes only) as the reference set. Upstream Regulator analysis predicts transcriptional regulators (TR) that can explain observed gene expression changes by measuring overlap between the dataset genes and the genes that are regulated by a TR using Fisher’s Exact Test and generating the “p‐value of overlap”.

### Identification of gene signatures with LASSO, PLSDA and Machine Learning

To select key features from 33 genes for classifying 4 groups (HC-NR, HC-R,HIV-NR,HIV-R), fit log2 transformed data to a multinomial logistic regression model via LASSO penalty using R package “glmnet”. 10-fold cross-validation (CV) was applied and the value of regularization parameter lambda giving minimum mean CV error (LamdaMin) was determined. The resulting model from LamdaMin was further assessed by Partial least square discriminant analysis (PLSDA) using R package “mixOmics” to identify minimum signature of gene features useful for differentiating 4 groups. Five genes identified by PLSDA with best classification ability for 4 groups were further assessed for their prediction ability by 4 machine learning algorithms (multiclass neural network, multiclass logistic regression, multiclass decision forest and multiclass decision jungle) using Microsoft Azure Machine Learning Studio (https://studio.azureml.net/). Cross-validation was performed by 3,5,10 folds. Four genes for classification of 2 groups (HIV,HC) were further assessed for their prediction ability by 4 machine learning algorithms (Two-class neural network, two-class logistic regression, two-class boosted decision tree and, two-class locally-deep support vector machine) using Microsoft Azure Machine Learning Studio. Cross-validation was performed by 3,5,10 folds.

## Supplementary information

Supplementary Figure and Tables
