# Supplementary material for: Single Cell Profiling Reveals PTEN Overexpression in Influenza-Specific B cells in Aging HIV-infected individuals on Anti-retroviral Therapy
Source: Sci Rep. 2019 Feb 21;9:2482. doi: 10.1038/s41598-019-38906-y (PMC6385500; doi:10.1038/s41598-019-38906-y)
Supplement: Supplementary file 1 — Supplementary Figure and Tables [file 41598_2019_38906_MOESM1_ESM.pdf]

SUPPLEMENTARY MATERIAL

Single Cell Profiling Reveals *PTEN* Overexpression in Influenza-Specific B cells in Aging HIV-infected individuals on Anti-retroviral Therapy

Lesley R. de Armas[1#], Suresh Pallikkuth[1#], Li Pan[1], Stefano Rinaldi[1], Nicola Cotugno[2,3] Sarah Andrews[4], Rajendra Pahwa[1], Adrian B McDermott[4], Paolo Palma[2], and Savita Pahwa[1]

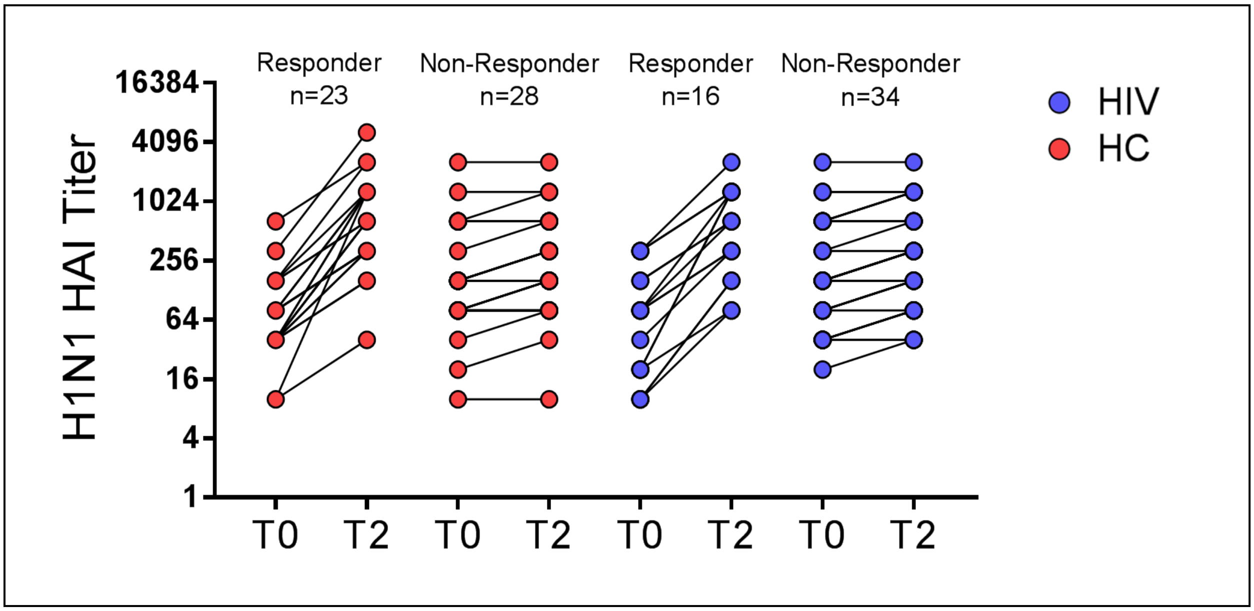

Figure S1. H1N1 HAI Titers for the Aging Influenza Vaccination Study. Lines connect individuals' titers from pre-vaccination (T0) to 21 days post-vaccination (T2) for Responders and Non-responders within HIV+ participants in blue and age-matched healthy controls in red.

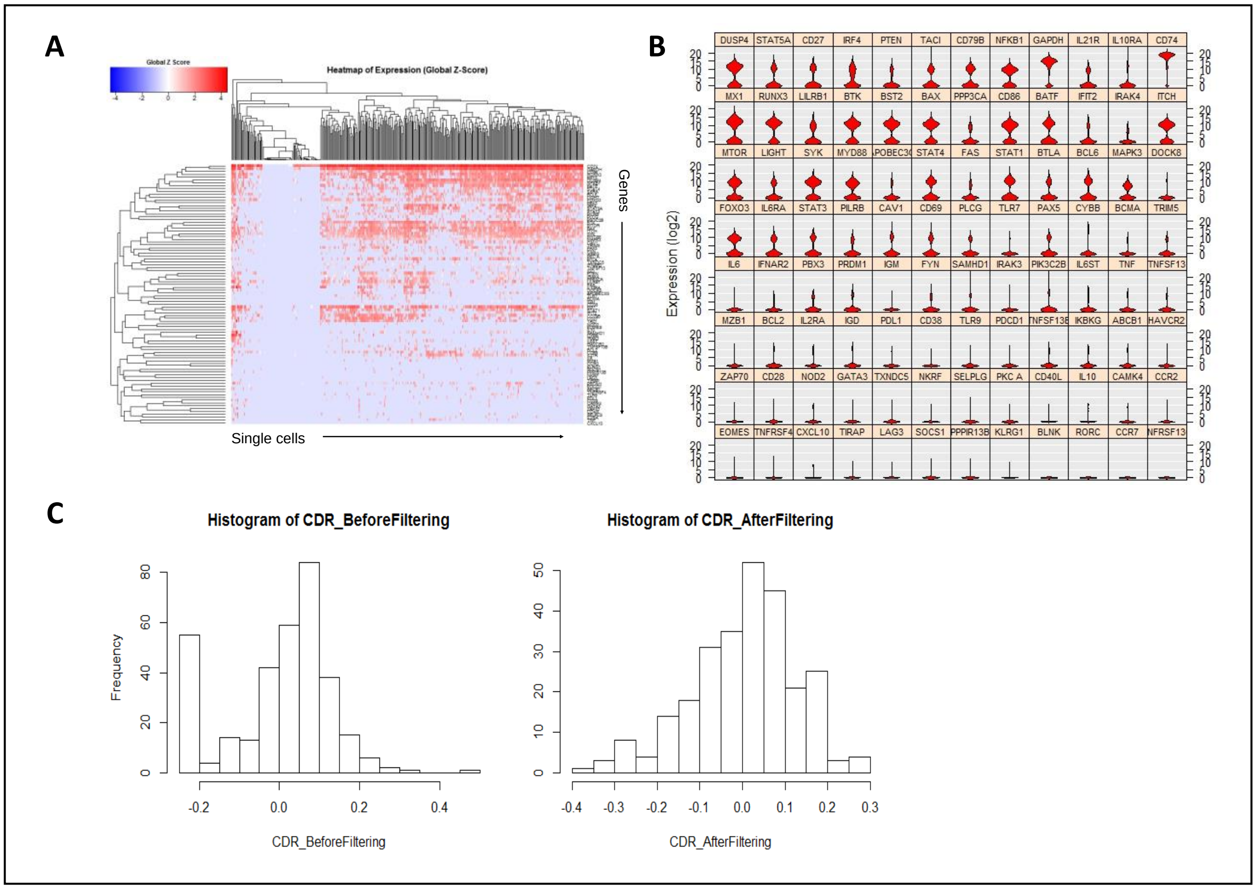

Figure S2. Single cell gene expression data from H1N1-specific B cells. A) Unsupervised hierarchical cluster heat map showing relative expression of all 96 genes (y-axis) in 360 single cells (x-axis) isolated from 12 individuals. B) Violin plot showing the distribution of expression for each gene in all cells. C) Single cell gene expression data distribution before and after CDR filtering.

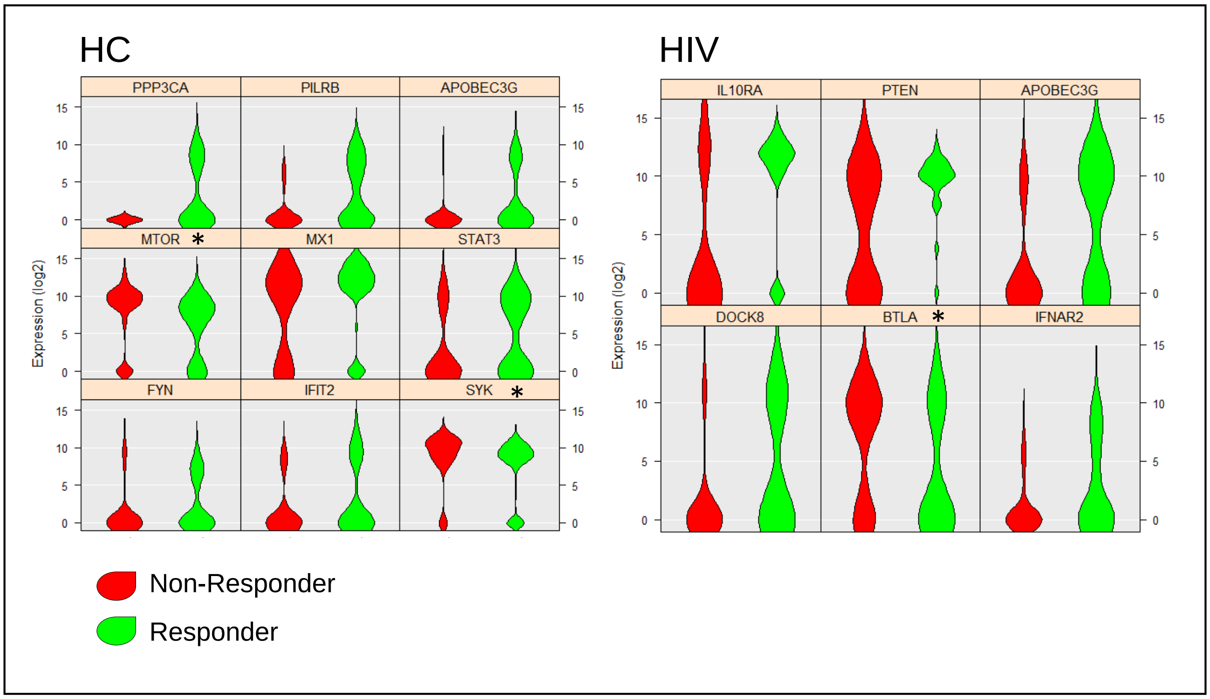

Figure S3. Gene signatures associated with Influenza vaccine response. Genes shown in the violin plots are differentially expressed between responders and non-responders with $p<0.05$. All genes were higher in responders unless the gene name is followed by *.

Table S1. List of Taqman Gene Expression Assays used in BioMark Experiments.

Gene	Taqman Assay	Gene	Taqman Assay	Gene	Taqman Assay	Gene	Taqman Assay
ABCB1	Hs00184500_m1	CYBB	Hs00166163_m1	IRF4	Hs01056533_m1	PRDM1	Hs00153357_m1
APOBEC3G	Hs00222415_m1	DOCK8	Hs00298892_m1	ITCH	Hs00395201_m1	PTEN	Hs02621230_s1
BATF	Hs00232390_m1	DUSP4	Hs01027785_m1	KLRG1	Rh00929962_m1	RORC	Hs01076112_m1
BAX	Hs00180269_m1	EOMES	Hs00172872_m1	LAG3	Hs00158563_m1	RUNX3	Hs00231709_m1
BCL2	Hs99999018_m1	FAS	Hs00531110_m1	LIGHT	Hs00998604_m1	SAMHD1	Hs00210019_m1
BCL6	Hs00277037_m1	FOXO3	Hs00921424_m1	LILRB1	Hs01848117_s1	SELPLG	Hs00380945_m1
BCMA	Hs00171292_m1	FYN	Hs00941600_m1	MAPK3	Hs00385075_m1	SOCS1	Hs00705164_s1
BLNK	Hs00179459_m1	GAPDH	Hs99999905_m1	MTOR	Hs00234508_m1	STAT1	Hs01013996_m1
BST2	Hs00171632_m1	GATA3	Hs00231122_m1	MX1	Hs00895608_m1	STAT3	Hs01047580_m1
BTK	Hs00975865_m1	HAVCR2	Hs00958623_m1	MYD88	Hs01573837_g1	STAT4	Rh02896026_m1
BTLA	Rh02889477_m1	IFIT2	Hs00533665_m1	MZB1	Hs00414907_m1	STAT5A	Rh02844611_m1
CAMK4	Hs00174318_m1	IFNAR2	Hs01022060_m1	NFKB1	Hs00765730_m1	SYK	Hs00374292_m1
CAV1	Hs00971716_m1	IGD	Hs00378878_m1	NKRF	Hs00938808_m1	TACI	Hs00963364_m1
CCR2	Hs00356601_m1	IGM	Hs01045555_m1	NOD2	Hs01550762_g1	TIRAP	Hs01568539_m1
CCR7	Hs00171054_m1	IKBKG	Hs00415849_m1	PAX5	Hs00277134_m1	TLR7	Hs00152971_m1
CD27	Hs00154297_m1	IL10	Hs00961622_m1	PBX3	Hs00608415_m1	TLR9	Hs00152973_m1
CD28	Hs00174796_m1	IL10RA	Hs00155485_m1	PDCD1	Hs00169472_m1	TNF	Hs00174128_m1
CD38	Hs01120071_m1	IL21R	Hs00222310_m1	PDL1	Hs00228839_m1	TNFRSF13C	Hs00606874_g1
CD40L	Hs00163934_m1	IL2RA	Hs00166229_m1	PIK3C2B	Hs00898518_m1	TNFRSF4	Hs00533968_m1
CD69	Hs00934033_m1	IL6	Hs00985639_m1	PILRB	Hs00273801_m1	TNFSF13	Hs00182565_m1
CD74	Hs00959498_g1	IL6RA	Hs00169842_m1	PKC A	Hs00925195_m1	TNFSF13B	Hs04234384_m1
CD79B	Hs01058826_g1	IL6ST	Hs00174360_m1	PLCG	Hs01008225_m1	TRIM5	Hs01552559_m1
CD86	Hs01567025_m1	IRAK3	Hs00936103_m1	PPP3CA	Hs00174223_m1	TXNDC5	Hs01046709_mH
CXCL10	Hs00171042_m1	IRAK4	Hs00928779_m1	PPPIR13B	Hs00367408_m1	ZAP70	Rh02837378_m1

Note: In green shading are gene inputs for predictive modeling.

Table S2. Summary of Results for machine learning algorithms and binary classification: HIV/HC

Model	AUC	CV 3 Fold	CV 5 Fold	CV 10 Fold
Two-class neural network	mean	0.890	0.883	0.897
Two-class neural network	standard error	0.030	0.025	0.056
Two-class locally-deep support vector machine	mean	0.892	0.880	0.883
Two-class locally-deep support vector machine	standard error	0.033	0.032	0.046
Two-class logistic regression	mean	0.872	0.864	0.871
Two-class logistic regression	standard error	0.034	0.027	0.054
Two-class boosted decision tree	mean	0.846	0.843	0.839
Two-class boosted decision tree	standard error	0.024	0.040	0.087
